# Morphometry Based on Effective and Accurate Correspondences of Localized Patterns (MEACOLP)

**DOI:** 10.1371/journal.pone.0035745

**Published:** 2012-04-23

**Authors:** Hu Wang, Yanshuang Ren, Lijun Bai, Wensheng Zhang, Jie Tian

**Affiliations:** 1 State Key Laboratory of Management and Control for Complex Systems, Institute of Automation, Chinese Academy of Sciences, Beijing, China; 2 Department of Radiology, Guang'anmen Hospital, Chinese Academy of Traditional Medicine, Beijing, China; 3 Life Science Research Center, School of Electronic Engineering, Xidian University, Xi'an, China; University Of Cambridge, United Kingdom

## Abstract

Local features in volumetric images have been used to identify correspondences of localized anatomical structures for brain morphometry. However, the correspondences are often sparse thus ineffective in reflecting the underlying structures, making it unreliable to evaluate specific morphological differences. This paper presents a morphometry method (MEACOLP) based on correspondences with improved effectiveness and accuracy. A novel two-level scale-invariant feature transform is used to enhance the detection repeatability of local features and to recall the correspondences that might be missed in previous studies. Template patterns whose correspondences could be commonly identified in each group are constructed to serve as the basis for morphometric analysis. A matching algorithm is developed to reduce the identification errors by comparing neighboring local features and rejecting unreliable matches. The two-sample *t*-test is finally adopted to analyze specific properties of the template patterns. Experiments are performed on the public OASIS database to clinically analyze brain images of Alzheimer's disease (AD) and normal controls (NC). MEACOLP automatically identifies known morphological differences between AD and NC brains, and characterizes the differences well as the scaling and translation of underlying structures. Most of the significant differences are identified in only a single hemisphere, indicating that AD-related structures are characterized by strong anatomical asymmetry. In addition, classification trials to differentiate AD subjects from NC confirm that the morphological differences are reliably related to the groups of interest.

## Introduction

The morphometric analysis of volumetric magnetic resonance images (MRI) of the brain has been widely applied by researchers to detect and quantify anatomical differences between subject groups, e.g., diseased and healthy brains [1]. Morphological differences could be used clinically to investigate the effects of pathology or treatment on anatomical structures [2], [3], [4]. They could also be used as biomarkers for neurodegenerative diseases (e.g. Alzheimer's disease) in computer-aided diagnosis [5], [6], [7].

Various morphometry methods have been developed in the past decade. One traditional way is to analyze morphological features from regions of interest (ROIs) with an *a priori* hypothesis [5], resulting in a wealth of findings pertaining to the particular ROIs. However, disease-related differences are sometimes abnormal from the ROIs and difficult to discover. In addition, abnormality spanning over multiple ROIs would result in inaccurate segmentation of the regions, therefore potentially reducing the reliability of morphometric analysis. These limitations can be effectively overcome by the morphometry methods that are based on voxel-wise measure, such as tissue density (VBM) [8] or deformation fields (DBM) [9]. Such methods assume that voxels could correspond between subjects via deformable registration and Gaussian smoothing. However, registration error still exists due to inter-subject variability, particularly in the highly variable cortices [10], and it is difficult to guarantee that images are not being over-aligned. Recently, morphometry methods based on adaptive regional elements [11], [12] have been proposed to improve the performance of traditional methods. The regional elements are automatically extracted from the training data in order to adapt to the pathology of interest, and thus *a priori* knowledge is not needed anymore. Moreover, the morphological information from the adaptive regional elements is more distinctive than voxels, hence reducing the registration errors. All the methods aforementioned make the same fundamental assumption that one-to-one correspondence could be achieved between subjects, that is, every corresponding unit for morphometric analysis, such as the voxel or regional element, can be identified in all subjects to represent the same anatomical structure. They neglect the fact that brain structures may exhibit distinct, multiple localized patterns across a population. Such localized patterns may only be partially present in subsets of subjects, and the one-to-one correspondence could not be achieved properly [13]. Thus the morphometric analysis might utilize the incorrect correspondences of localized patterns. Given that the sample size of training subjects is often limited, incorrect correspondences would seriously disturb the distribution of morphological features, and further reduce the reliability of the morphometric analysis.

Feature-based morphometry (FBM) has been recently proposed for the situation where the one-to-one correspondence of localized patterns is ambiguous or difficult to achieve [13]. Two issues are taken into consideration: what can be utilized to represent the localized patterns for the purpose of eliminating incorrect correspondences, and how statistical morphometric analysis can be computed to identify morphological differences from the partially present patterns. Local features have been used to represent the localized patterns in previous studies [13], [14]. Local feature are salient image regions with high repeatability of detection [15], so they are used to identify localized patterns of the same underlying structure in different subjects. Local features could also be very distinctive in terms of their locations and high-dimensional appearance descriptors [16]. Thus incorrect correspondences arising from different underlying patterns could be eliminated to some extent. Moreover, local features could be extracted in a scale-invariant manner using the scale-invariant feature transform (SIFT) [17], [18]. SIFT has been developed based on the scale-space theory to handle image structures at different scales [19], and widely used in the computer vision applications such as object recognition[20], robotic mapping and navigation [21], and action recognition [22]. In the scale-invariant manner, localized patterns are described at their characteristic scales, rather than at arbitrary scales such as voxel-level. Their correspondences could hence be robustly identified despite that their scales vary between subjects. In order to identify morphological differences from the partially present patterns, FBM constructs a probabilistic model on clusters of local features, and quantifies the statistical regularity between localized patterns and groups by occurrence likelihood [13], [14]. The method has shown good performance in identifying group-related structures with different occurrence likelihoods. However, it can not characterize what kind of specific morphological differences has happened to the structures, e.g., atrophy or enlargement as shown in traditional morphometry method. This is because the correspondences for the same localized patterns are often sparse and only present in a minority of the training subjects [13]. The sparse correspondences are not effective to reflect distributions of underlying anatomical structures, making it unreliable to evaluate the specific morphological differences between two groups. On the other hand, localized patterns are found to be more commonly present than they are detected. Their correspondences are actually missed as the same underlying patterns in many subjects are not detected as local features. Therefore, denser detections of local features are required so as to improve the effectiveness of the correspondences for a specific morphometric analysis.

Although correspondences between subjects are identified according to distinctive local features, two types of error should be taken into consideration and reduced. False positives (FP) occur when local features arising from different underlying anatomical structures are accepted as correspondences, while false negatives (FN) occur when local features arising from the same structure are rejected as non-correspondences [13]. Matching techniques that identify correspondences of local features between images have been proposed to reduce error rates of both FP and FN [17]. More precisely, in each subject, the best candidate correspondence of a localized pattern is selected by identifying its nearest neighbor. The nearest neighbor of a localized pattern is defined as the local feature with the minimum Euclidean distance of the appearance descriptors. Then, unreliable candidates that are likely to arise from different underlying structures are discarded. Global thresholds on distances to the nearest neighbor do not perform well because the range of inter-subject variability is different as the patterns of different structures vary [23]. An efficient algorithm has been proposed based on the ratio of neighbor distances, assuming that a good correspondence should make a significant difference between the distance of the nearest neighbor and the distance of the second-nearest neighbor [17]. The algorithm rejects a match if the ratio of the nearest distance to the second-nearest distance surpasses a given threshold. Such a ratio-based matching algorithm performances well because incorrect matches often have similar distances with a number of other neighbors. However, for situations when local features are densely detected, the nearest two neighbors overlap with each other and arise from the same underlying structures, causing the distance ratio to become higher. Correct matches could thus be rejected and the error rate of FN may be increased.

This paper presents a new method, named Morphometry based on Effective and Accurate COrrepsondences of Localized Patterns (MEACOLP), in order to overcome certain limitations in the previous morphometric analysis. The scale-invariant feature (SIF) is used to represent localized patterns that may be partially present in a population, and the morphometric analysis is based on the correspondences of localized patterns. The emphasis of this paper is to identify effective and accurate correspondences for a specific morphometric analysis. Since the correspondences lost in FBM [13] are mainly due to the detection procedure, the effectiveness of correspondences is improved by enhancing the detection repeatability of local features. This is achieved by a novel two-level scale-invariant feature transform (2L-SIFT) which extracts denser secondary SIF sets with relaxed constraint. The missed local features in standard detection procedure could be thus recalled by the 2L-SIFT. Template patterns are generated from the SIF sets to serve as the basis for morphometric analysis, rather than the local feature clusters in FBM [13]. Their correspondences are expected to be identified in most subjects according to the distribution of SIFs in training data. In order to identify accurate correspondences for the template patterns, the ratio-based matching algorithm is modified to adapt to the dense secondary set. Specifically, the algorithm identifies correspondences by first investigating all spatially neighboring SIFs, rather than the nearest two, and then rejecting unreliable correspondences with thresholds that are related to overlap extent of the neighbors. Accordingly, it could enhance the accuracy of correspondences by not only rejecting incorrect correspondences but also selecting better correspondences from overlapped SIFs that arise from the same underlying structures. Morphological features are then extracted from the correspondences of the template patterns using the scale-space parameters, and the two-sampled *t*-test is lastly performed to detect and quantify morphological differences between groups. According to the scale-space parameters, template patterns with significant morphological features would be characterized as the translation or scaling of the underlying structures. For validation, the analysis of Alzheimer's disease (AD) is used to demonstrate the morphometry method.

## Methods

This section describes the new MEACOLP method that is based on effective and accurate correspondences of localized patterns. MEACOLP begins with a set of subject images that have been spatially normalized via global linear registration. The registration aims to approximately align potential corresponding structures in different subjects. A geometry constraint could thus be applied to identify correspondences for underlying structures more precisely. The method first models each subject image as two sets of SIFs using the 2L-SIFT. Template patterns are then generated from the SIFs that are representative according to the training data. Afterwards, morphological features are extracted from correspondences of the template patterns for morphometric analysis.

### 2.1 Two-level scale-invariant feature transform (2L-SIFT)

The 2L-SIFT aims to improve the density of SIFs detected by the standard SIFT procedure. Sparse SIFs are not effective to reflect specific morphological differences because as a matter of fact some SIFs for the same underlying structures are lost [13]. The SIF-lost phenomenon comes from the fact that extrema in discretely sampled scale-space are the precondition of SIFs [17], [18]. The underlying anatomical structures vary in geometry and appearance from one subject to another. The extrema in the discrete scale-space may thus vanish in some subjects, and the SIFs based on these extrema would disappear. To deal with the SIF-lost issue, the 2L-SIFT attempts to enhance the detection repeatability by redefining the constraint for extrema. It consists of construction of the difference-of-Gaussian (DoG) function, selection of two-level candidates, candidate adjustment and description.

#### 2.1.1 Construction of the DoG function

According to the scale-space theory, the scale-normalized Laplacian of the Gaussian is required for true scale-invariance, and its extrema produce the most stable image features compared to a range of other possible image functions [17], [19]. In practice for a volumetric image, the scale-normalized Laplacian of the Gaussian is approximated by the four-dimensional DoG function 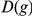 which is expressed as:
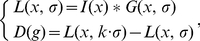
(1)where *x* denotes the coordinate vector; and 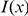 represents the original image, 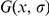 the Gaussian kernel with variable-scale 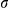, and 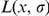 the discrete scale-space of the original image; *k* denotes a constant factor for the discrete sampling of the scales, and 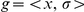 a geometry vector in the scale-space.

#### 2.1.2 Selection of two-level candidates

**Figure 1:**
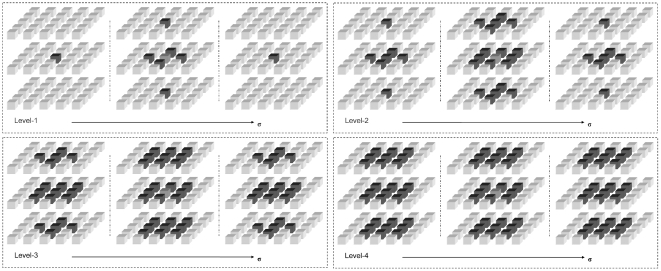
Four levels of constraints for the extrema detection in discrete scale-space. The neighboring voxels for judging whether or not a voxel (the central) is an extremum are shown in black. Level-1 to level-4 constraints consist of the closest 8, 32, 64 and 80 voxels, respectively.

According to the scale-space theory, the extrema of the DoG function could be selected as candidates for SIFs. An extremum in 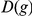 is determined by the standard SIFT if it is a local maximum or minimum in its 3×3×3×3 discrete neighborhood, as shown in Fig. 1 (4). Such an extremum may vanish due to disturbances from inter-subject variability, leading to the SIF-lost phenomenon. It is noted that the discrete neighborhood functions as a constraint of the extremum. A wider neighborhood would strengthen the constraint and reduce the number of extrema. Given that closer voxels are more correlative, the disturbances mostly affect extrema via farther voxels in the neighborhood. Accordingly, weakening the constraint to a more compact neighborhood provides a solution to reducing the disturbance from inter-subject variability and increasing the detection repeatability.

The voxels in the 3×3×3×3 neighborhood are divided to generate different levels of constraints according to the spatial radius. The spatial radius *r* is defined as the maximum Euclidean distance to the central voxel in the neighborhood. The level-R constraint denotes that the neighborhood is composed of voxels satisfying 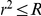, where *R* = 1, 2, 3, 4, as shown in Fig. 1. In 2L-SIFT, only two of the four levels are select. Level-4 constraint, which is the strictest, is used to extract a main candidate set for SIFs which are very stable in scale-space. On the other hand, the level-1 constraint, which is the lightest, is used to extract a secondary candidate set for SIFs which are much denser than the main set.

#### 2.1.3 Candidate adjustment and description

Points in the candidate sets are discrete in scale-space, and it is pointed out that refining these points to a sub-voxel level would improve the stability for matching local features [17]. Given that the four-dimensional interpolations used for the standard SIFT could not be used with solely the voxels from the level-1 constraint, a refinement in 2L-SIFT is performed by one-dimensional interpolation. Moreover, two kinds of unstable points in the candidate sets are identified and discarded to further improve the stability. They are the low contrast points which are sensitive to image noise and the edge points which are located unsteadily on the edge of image content (e.g. tissue surface) [17]. After that, each candidate is described as a SIF 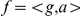 in terms of a geometry vector 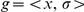 and an appearance descriptor *a*. The geometry vectors are assigned the refined values of interpolation, and the appearance descriptors are resampled from voxel intensities in a cubical region centered on the geometry vectors [13].

Given a group of training subjects denoted as 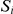, the 2L-SIFT procedure models each subject as two sets of SIFs: a main set of SIFs 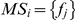 which are detected with the level-4 constraint to represent more stable anatomical patterns, and a secondary set of SIFs 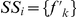 detected with the level-1 constraint to extract denser localized patterns.

### 2.2 Generating template patterns

Template patterns serve as potential group-related patterns for morphometric analysis. They are generated according to the distribution of the SIFs from the training data. Several issues are taken into consideration. Firstly, group-related patterns may occur in all of the anatomical structures with different locations and sizes. All stable SIFs in the main sets would be hence treated as candidates to construct the template patterns. Secondly, template patterns should be representative in each group in order to effectively reflect the distribution of potential group-related patterns. Correspondences of the candidate patterns would be identified from the training images to evaluate the performance of candidates without bias. Thirdly, the template patterns should be distinctive in order to identify accurate correspondences from the same underlying structures as well as reject incorrect correspondences from different structures. This requires a comprehensive description of the template patterns, including the geometry, appearance, and the variability learnt from the training data. We will present in detail the automatic procedure for generating template patterns as follows.

#### 2.2.1 Identification of correspondences

For a candidate 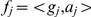, its correspondence in each subject is identified from the secondary set. Since potential correspondences have been approximately aligned with a registration step, a geometry constraint is first applied to obtain a geometrical neighbor set as:
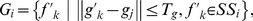
(2)where 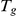 is the geometry threshold. This threshold is related to the maximum registration error and is empirically set to 10 voxels in our study. The correspondence is then identified according to the appearance descriptors. The ratio-based matching algorithm [17] is modified to adapt to the dense SIFs in the secondary set. The modified algorithm investigates all spatially neighboring SIFs, and makes a decision based on thresholds that are adjusted by the overlap extent of the neighboring SIFs. Let 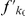 represent the nearest neighbor in the geometrical neighbor set, and 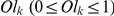 represent the extent of the overlap between 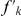 and 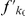. Then, the correspondence is identified as:
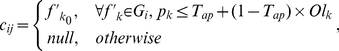
(3)where *null* denotes the rejection of the correspondence, and 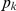 a ratio of the Euclidean distances from the nearest neighbor to another neighbor; 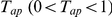 is an initial ratio-based threshold and is empirically set to 0.8. 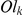 is computed as the percentage of the volume of overlapped description region.

As a result, each candidate is associated with a set of correspondences which could be seen as a cluster in the descriptor space. In order to further reduce FP, outliers in each cluster are eliminated based on the distribution of the correspondences which is expected to follow a normal distribution. We compute the Euclidean distances from the correspondences to the gravity center of their cluster, and set a maximum permissible distance 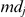 as:
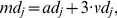
(4)where 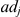 and 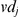 denote the average and standard deviation of the distances, respectively. The maximum permissible distance denotes the confidence interval that is expected to include most of the correspondences arising from the same underlying patterns.

#### 2.2.2 Generating representative within-group patterns

The sizes of the correspondence sets are used to evaluate the effectiveness of the candidate, and the maximum permissible distances are used to evaluate the accuracy of the correspondences of the candidate. Based on the two measures, we discard the ineffective candidates whose correspondences are identified in no more than a half of the subjects, and select representative candidates from overlapping candidates which arise from the same underlying anatomical structures. Here, two candidates are determined to be overlapping if one candidate falls in the cluster range of the other.

The representative candidates and their correspondences represent the patterns arising from the same anatomical structures in a group, and form compact clusters in the space of descriptors without overlap. Representative within-group patterns are then described as:
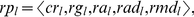
(5)where 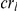 denotes the correspondence rate defined as the percent of the subjects where correspondences of the same underlying patterns are identified; 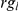 and 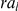 specify the average geometries and appearance descriptors of the correspondences, respectively; 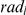 denotes the average distances from the correspondences to 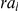, and 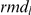 the maximum permissible distance as in formula (4).

#### 2.2.3 Constructing the template

The template patterns are constructed using the comparable patterns that are representative within each group. Given *RpSet(A)* and *RpSet(B)* denoting the representative pattern set in group A and B, respectively, the template patterns are denoted as:
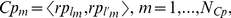
(6)where 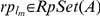, 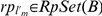, and 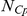 denotes the total number of template patterns. The comparable patterns could be identified using a bi-directional matching algorithm which is also ratio-based as described in formula (3); however, it accepts 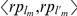 if and only if the match of 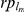 is identified as 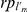 and the match of 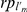 is identified as 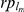.

### 2.3 Extracting morphological features

In this step, we extract morphological features for each subject based on template patterns. This is achieved by firstly identifying correspondences of the template patterns for each subject, and then assigning specific morphological features to the correspondences. For each template pattern 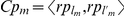, its correspondence 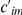 in subject 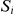 is identified as:
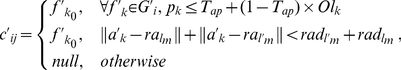
(7)where 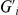 is the neighbors set computed under the Constraint of Geometry (*CoG*) and the Constraint of Appearance (*CoA*) defined in formula (8); 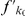 is the nearest neighbor. In formula (7), 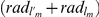 denotes the average distance from correspondences to the template pattern, and it is used to recall false negatives of the ratio-based matching algorithm.
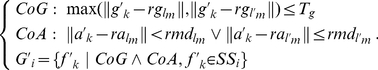
(8)


Geometry parameters in terms of locations and scales are used to describe each correspondence since they are stable patterns in scale-space. The features 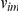 for correspondence 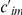 are computed as:
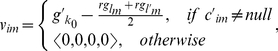
(9)where 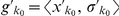 denotes the geometry of a non-null correspondence. As a result, a vector with morphological features is extracted for each subject.

### 2.4 Morphometric analysis

The analysis aims to detect and quantify significant differences between groups of morphological features. A one-sided, two-sample *t*-test is used to assess the significance of the feature difference. The *P*-value measures the degree of association between an individual feature and the group of interest, e.g. Alzheimer's disease. Morphometric features can be sorted according to the *P*-values to identify the anatomical structures most indicative of the group. In order to detect reliable group-related features, a false discovery rate (FDR) control is used to correct *P*-values for multiple comparisons to control the probability of committing type I errors. Considering that the morphological features are extracted from the scale-space parameters in terms of locations and scales, each morphological difference is characterized as a translation or scaling of its corresponding template pattern.

### 2.5 Classifying new subjects

The MEACOLP framework has a potential to support computer-aided diagnosis. More precisely, MEACOLP can be used to identify morphological difference in new subjects and to classify new subjects according to two groups of training subjects. Classification of a new subject image begins with aligning the image via the same transformation approach as the training images. SIFs with the levle-4 constraint are then extracted, and their correspondence sets are identified from the training subjects, and effective sets with correspondences present in most training subjects are selected. After that, a naive Bayes classifier is applied under the assumption of conditional feature independence as described in [13]. The classification is primarily driven by the following data likelihood ratio (*DLR*):
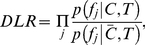
(10)where *f_j_* are the SIFs with effective correspondence sets, *C* the group label in the training data and *T* the transform of alignment. Given a threshold, the *DLR* value above the threshold indicates that the new subject belongs to group *C*. The threshold of *DLR* can be adjusted to balance the sensitivity and specificity of classification in clinical settings. Here, the conditional probability *p(f_j_|C,T)* is estimated using the kernel density estimation which is expressed as:
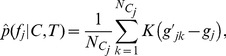
(11)where *K* is the Gaussian kernel function, 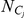 the size of the effective set, and 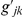 the geometry vectors of the correspondences in the effective set.

## Results

### 3.1 Materials

In order to validate the performance of MEACOLP, we performed experiments on a large, publicly available, cross-sectional dataset in the OASIS project [24] (http://www.oasis-brains.org/). The dataset includes MRI data from 100 probable Alzheimer's disease (AD) subjects and 98 normal control (NC) subjects. The AD subjects are diagnosed clinically from very mild to moderate dementia characterized by the Clinical Dementia Rating (CDR) scales [25]. Subjects in the dataset are all right-handed, with ages ranging from 60 to 96 years. The two-sample *t*-test (*DoF* = 196) indicates there are no significant differences (*t* = 0.73, *P* = 0.47) between the age distributions for the NC group (75.92±8.99) and the AD group (76.76±7.12). For each subject, at least three T1-weighted magnetization-prepared rapid gradient echo (MP-RAGE) images have been obtained according to the following protocol: 128 sagittal slices, matrix = 256×256, TR = 9.7 ms, TE = 4 ms, flip angle = 10°, and resolution = 1 mm×1 mm×1.25 mm. Moreover, the images have been gain-field-corrected and averaged in order to improve the signal-to-noise ratio. Images from all subjects have been aligned within the Talairach reference frame (voxel size = 1×1×1 mm^3^) via the affine transform, and the skulls have been masked out in the OASIS project [24]. We then adjusted abnormal voxel intensities to normal levels via histogram analysis in order to make all images in similar intensity range, i.e., lower the intensities of the top 0.05% voxels that may arise from the residual skulls. Subsequently, we normalized the range of voxel intensity to [0, 1] for all images. The 2L-SIFT was then applied to extract a main SIF set and a secondary SIF set for each image. An analysis of the Hessian matrix was used in 2L-SIFT to identify and discard edge points from the SIF sets, as described in the previous study [13]. However, the threshold in the analysis was lowered, since it was excessively strict for our method. As a result, averagely 1300 SIFs were extracted from each image for the main set, and 5100 SIFs for the secondary set.

### 3.2 Template patterns

**Figure 2:**
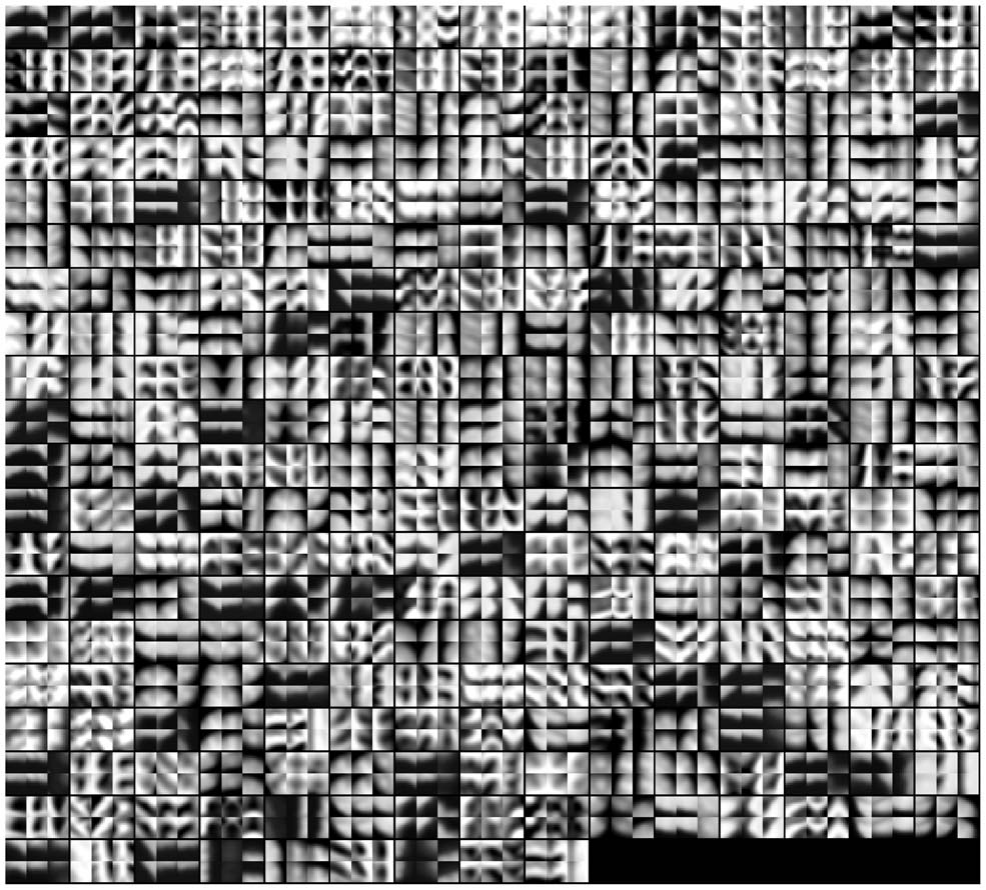
Template patterns. Each template pattern consists of two representative within-group patterns, shown in a grid with the central sagittal, coronal, and axial slices. In each grid, the upper and the lower slices are from the representative patterns of the Alzheimer's disease group and the normal control group, respectively.

On the training subjects, MEACOLP generated 408 representative patterns in the AD group and 482 in the NC group, and constructed 294 template patterns, as shown in Fig. 2. According to the training data, correspondences of the template patterns were expected to be identified in most of the subjects in each group.

**Figure 3:**
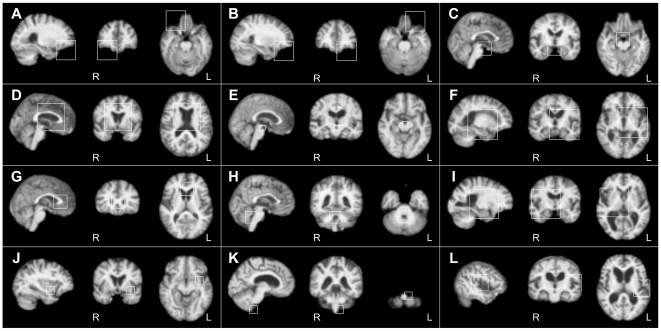
Correspondences of the twelve most stable template patterns. They are illustrated in subject images with the sagittal, coronal, and axial slices. The squares indicate 3D regions for the descriptors of the correspondences. L = left; R = right.

In order to further illustrate the template patterns, we list the twelve most stable template patterns in Fig. 3 in terms of their corresponding regions in subject images. These template patterns have the most correspondences in both groups and the most compact clusters in the descriptor space. Some template patterns are approximately symmetrical in pairs in Fig. 3. For instance, patterns (A, B) denote the right and left 3D corner regions in front of the temporal poles and beneath the orbitofrontal cortex. Patterns (F, I) denote two big regions in the left and right hemispheres, respectively. They are centered at the posterior limbs of the right and left internal capsules, mainly including the internal capsules, thalamus, basal ganglia, and insular lobe. Note that there might be some localized differences between symmetrical template patterns. For example, while pattern (F) centers on the left of the body of the third ventricle, its symmetrical pattern (I) centers on the right of the taenia thalami, a little more anterior and upper than (F). Five template patterns are centered between the two hemispheres. Pattern (C) denotes the 3D corner in front of the pons and between the left and right parahippocampal gyrus, pattern (D) the region including anterior parts of both lateral ventricles, pattern (E) the cisterna interpeduncularis, pattern (G) the genu part of the corpus callosum, and pattern (H) the fourth ventricle. There are three unilateral template patterns whose contralateral patterns were not identified in the twelve most stable template patterns, namely pattern (J) denoting a region of the external capsule near the claustrum, pattern (K) the 3D corner on the left of the medulla oblongata and beneath the left cerebellum, and pattern (L) a corner region of the left lateral sulcus.

### 3.3 Comparison between 2L-SIFT and standard SIFT

**Figure 4:**
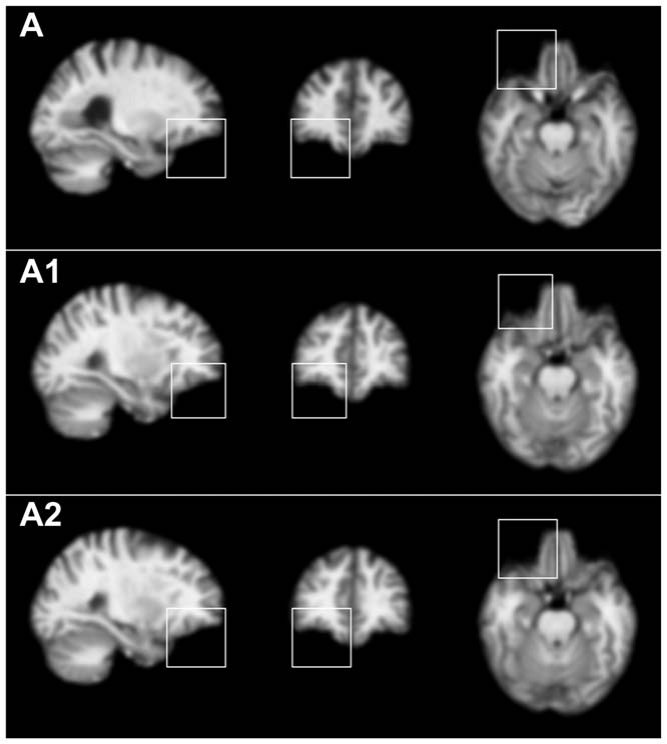
More accurate correspondences. The squares indicate 3D regions for the descriptors of localized patterns. A is a template pattern shown in subject image. A1 and A2 show two local features extracted from the same training subject, both arising from the same underlying anatomical structure as A. A2 is a more accurate correspondence than A1 according to the location and the scale.

The standard SIFT models each volumetric image as a main set of SIFs. In contrast, the novel 2L-SIFT additionally extracts a denser secondary set of SIFs with a relaxed constraint. In the experiment, 2.61×10^5^ SIFs were extracted for the main sets and 1.01×10^6^ SIFs for the secondary sets from all the 198 training images. The 2L-SIFT are proposed to recall the local features that may be missed by the standard SIFT. Furthermore, 2L-SIFT could be used to identify more accurate correspondences by selecting more similar SIFs from the secondary set, as illustrated in Fig. 4. For the underlying anatomical structure of template pattern (A), the standard SIFT extract a SIF (A1) while the 2L-SIFT extract (A1) and (A2). We can find that (A2) is a better and more accurate correspondence than (A1) by comparing their appearance distances to the template pattern. We compared the 2L-SIFT with the standard SIFT according to the effectiveness and accuracy of their correspondences. Correspondences for each template pattern were identified from the main sets and the secondary sets, respectively. The correspondence rates were computed and used to measure the effectiveness. The distribution of the appearance distances from correspondences to the template pattern, expressed as mean±standard deviation, were computed and used to measure the accuracy. Table 1 shows the results for the twelve most stable template patterns. The correspondence rates from the 2L-SIFT are higher that those from the standard SIFT for most of the template patterns, demonstrating that more effective correspondences have been identified for potentially group-related patterns. All distances from the 2L-SIFT are not larger than those from the standard SIFT, revealing that the effectiveness has been improved without losing any accuracy. Moreover, most distances from the 2L-SIFT are in fact a bit smaller, indicating that the correspondences identified by 2L-SIFT are more accurate. In some cases, a trade-off was achieved between effectiveness and accuracy. For example, the correspondence rate of the 2L-SIFT for template pattern (K) is slightly lower than that of the standard SIFT in the NC group; however, the correspondences are more accurate according to the distances.

**Table 1:** Comparison of correspondences between 2L-SIFT and SIFT.

	2L-SIFT			Standard SIFT		
No.	CR (AD)	CR (NC)	DIST	CR (AD)	CR (NC)	DIST
A	94%	98%	0.08±0.02	77%	78%	0.08±0.02
B	97%	96%	0.09±0.02	86%	89%	0.10±0.02
C	94%	93%	0.18±0.05	41%	40%	0.19±0.06
D	97%	87%	0.33±0.10	96%	88%	0.34±0.10
E	92%	92%	0.22±0.06	93%	88%	0.28±0.07
F	93%	90%	0.26±0.07	62%	32%	0.27±0.06
G	95%	94%	0.34±0.10	91%	92%	0.34±0.10
G	92%	92%	0.23±0.06	89%	92%	0.25±0.08
H	94%	87%	0.25±0.07	77%	49%	0.28±0.08
I	92%	96%	0.37±0.07	87%	92%	0.38±0.07
J	88%	92%	0.22±0.06	84%	82%	0.23±0.07
K	84%	91%	0.34±0.08	88%	82%	0.38±0.09

The twelve most stable template patterns were used for the comparison. The corresponding rates (CR) for both the Alzheimer's disease group (AD) and the normal control group (NC) demonstrate the improvement of the effectiveness of the 2L-SIFT. The appearance distances (DIST) in terms of mean ± standard deviations demonstrate the improvement of accuracy of the 2L-SIFT.

### 3.4 Morphological differences

**Figure 5:**
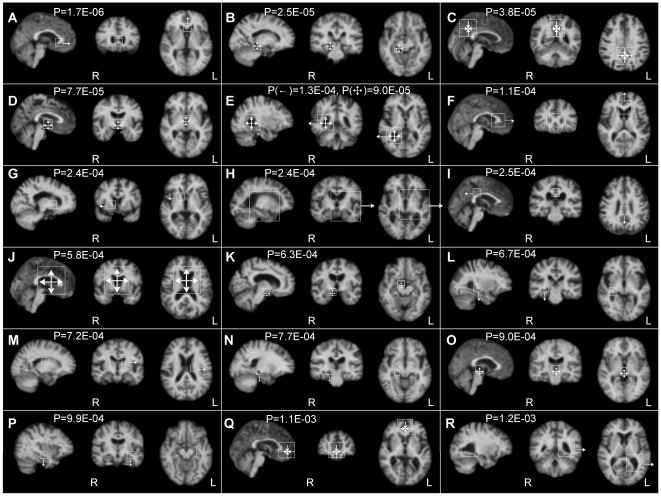
Group-related template patterns. The patterns are illustrated in subject images with the sagittal, coronal, and axial slices. The squares indicate the 3D regions for the descriptors of the correspondences. The arrows demonstrate the morphological differences of AD in terms of translation or scaling. L = left; R = right.

As described in section 2.3, we extracted morphological features from the correspondences of the template patterns. There were 291 template patterns with correspondences identified in most subjects of both the AD and NC groups. A 1164-dimensional feature vector was extracted for each subject using the four-dimensional scale-space parameters. The one-sided, two-sample *t*-test was performed to assess the statistical significance of the relationship between an individual feature and subject groups. There are 28 morphological features identified as significantly group-related (*P*<0.05, FDR corrected). For these 28 features, 26–27 could thus be expected to result from valid group-related anatomical structures. We illustrated 19 example features in terms of specific modifications of the corresponding template patterns in Fig. 5, and listed their *P*-values in Table 2.

**Table 2:** Statistics of the group-related template patterns.

	*P*-value			
No.	*x_1_*	*x_2_*	*x_3_*	*x_4_*
A	3.9E-01 (↑)	**1.7E-06** (↓)	7.0E-03 (↑)	2.2E-01 (↓)
B	1.3E-01 (↓)	2.0E-01 (↓)	9.1E-02 (↓)	**2.5E-05** (↑)
C	1.0E-01 (↓)	4.3E-01 (↑)	2.8E-01 (↑)	**3.8E-05** (↓)
D	2.8E-01 (↑)	3.9E-02 (↑)	2.8E-01 (↑)	**7.7E-05** (↑)
E	**1.3E-04** (↓)	1.7E-02 (↑)	6.2E-03 (↓)	**9.0E-05** (↑)
F	4.3E-01 (↓)	**1.1E-04** (↓)	4.5E-01 (↑)	3.9E-01 (↓)
G	**2.4E-04** (↓)	4.1E-01 (↓)	5.0E-01 (↑)	3.0E-01 (↑)
H	**2.4E-04** (↑)	4.1E-01 (↓)	2.8E-01 (↓)	4.9E-01 (↑)
I	5.0E-01 (↓)	**2.5E-04** (↑)	2.4E-01 (↓)	4.7E-01 (↑)
J	3.1E-01 (↓)	3.8E-01 (↓)	8.6E-02 (↑)	**5.8E-04** (↑)
K	7.7E-03 (↓)	4.3E-01 (↓)	4.2E-01 (↓)	**6.3E-04** (↑)
L	9.8E-03 (↓)	5.4E-02 (↓)	**6.7E-04** (↓)	1.4E-01 (↑)
M	**7.2E-04** (↑)	2.8E-01 (↓)	2.8E-01 (↑)	3.3E-01 (↑)
N	1.0E-01 (↓)	1.0E-01 (↓)	**7.7E-04** (↓)	1.3E-02 (↑)
O	1.1E-01 (↑)	2.6E-02 (↓)	3.7E-01 (↓)	**9.0E-04** (↑)
P	3.4E-01 (↑)	3.1E-01 (↓)	**9.9E-04** (↓)	1.5E-03 (↓)
Q	4.4E-01 (↑)	4.8E-02 (↓)	4.4E-02 (↓)	**1.1E-03** (↓)
R	**1.2E-03** (↑)	5.5E-02 (↑)	2.4E-02 (↓)	3.1E-02 (↑)

One-sided, two-sample *t*-tests were applied to each individual feature. Features *x_1_*, *x_2_*, *x_3_* and *x_4_* denote the four-dimensional scale-space parameters. For each feature, the up arrow indicates (AD>NC) and the down arrow indicates (AD<NC*P*<0.05, FDR corrected) are shown in bold.

The group-related template patterns are consistent with brain regions known to differ between the AD and NC groups, involving enlargement of ventricles and atrophy of cerebral cortex. Eight template patterns were modified due to AD in terms of scaling, indicating atrophy or enlargement of their underlying anatomical structures. Patterns (B, K) demonstrate enlargement of the extracerebral space adjacent to the right hippocampal sulcus, reflecting atrophy of different parts of the right parahippocampal gyrus [26]. Pattern (C) demonstrates atrophy of a cortical region which includes the dorsal posterior cingulate gyrus and the precuneus of the parietal lobe in both hemispheres [26], [27]. Patterns (D, O, E, J) demonstrate enlargement of the anterior and the posterior parts of the third ventricle, the posterior body of the right ventricle, and the anterior parts of both lateral ventricles, respectively. The enlargement of ventricle system reflects atrophy of the surrounding structures [28], [29]. Pattern (Q) demonstrates atrophy of the anterior cingulate cortex and the anterior prefrontal cortex [26], [30]. Eleven template patterns were modified in terms of translation, indicating atrophy or enlargement of their adjacent structures. Patterns (A, F) demonstrate forward shifting of the rostrum and the genu of the corpus callosum, reflecting enlargement of the anterior parts of lateral ventricles [28] and atrophy of the anterior cingulate gyrus [12], [31]. Patterns (E, R) demonstrate rightward and leftward shifting of the center of the two posterior bodies of the lateral ventricles, reflecting symmetrical enlargement of the ventricles and atrophy of the adjacent temporal cortex. Pattern (G) demonstrates rightward shifting of the anterior limb of the right internal capsule, reflecting enlargement of the anterior part of the right lateral ventricle [28]. Pattern (H) demonstrates leftward shifting of the posterior limb of the left internal capsule, reflecting enlargement of the third ventricle [29] and atrophy of the adjacent insular cortex [26], [32]. Pattern (I) demonstrates backward shifting of a posterior part of the callosal sulcus, reflecting the atrophy of the posterior cingulate gyrus [27]. Patterns (L, P) demonstrate downward shifting of the right posterior temporal stem and the left anterior temporal stem, reflecting atrophy of the right hippocampus and the parahippocampal gyrus [26], [33]. Pattern (M) demonstrates leftward shifting of the left internal capsule around the body of the lateral ventricle, reflecting enlargement of the left ventricle [28]. Pattern (N) demonstrates downward shifting of the extracerebral space around the right hippocampal sulcus, reflecting atrophy of the right parahippocampal gyrus [26].

Spatially neighboring template patterns arising from different anatomical structures are often modified by the same factors, resulting in consistent morphological changes. For example, the four patterns (A, F, J, Q) all demonstrate enlargement of the anterior lateral ventricles and atrophy of anterior cingulate gyrus, and the four patterns (B, K, L, N) demonstrate the atrophy of the right parahippocampal gyrus, and the three patterns (D, H, O) demonstrate the enlargement of the third ventricle.

### 3.5 Classification

MEACOLP can be used to classify new subjects in a computer-aided diagnosis scenario. As suggested by Toews [13], we took into account the clinical and demographic information of the experimental subjects so as to illustrate the effects of age and the severity of clinical diagnosis on classification performance. Three different divisions of the OASIS subjects were used as follows.

1. Subjects aged 60–80 years, CDR = 1 (66 NC, 20 AD);
2. Subjects aged 60–96 years, CDR = 1 (98 NC, 28 AD), to illustrate classification of elderly subjects;
3. Subjects aged 60–80 years, CDR = 0.5 and 1 (66 NC, 70 AD), to illustrate classification of very mild AD.

**Figure 6:**
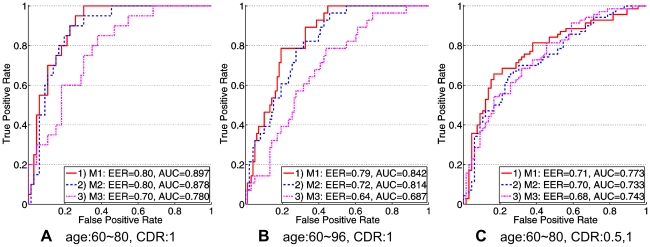
ROC curves for three methods on three different divisions. M1, M2, and M3 denote the proposed method, Toews' method, and the proposed method without the secondary set, respectively. M1 outperforms M2 a little, and performs much better than M3. Including wider range of age (B) or severity of clinical diagnosis (C) both result in reduced classification performance.

On these divisions, we compared our method (M1) to the Toews' method [13] (M2). In order to analyze the effect of the secondary SIF set proposed in this paper, we designed a classification procedure (M3) which is the same as our method except the SIF sets for correspondences. In M3, the correspondences were identified from the main SIF sets instead of the proposed secondary sets. Classification was performed in a leave-one-out manner, where each test subject in turn was kept aside and classified according to all other subjects as training subjects. The receiver operating characteristic (ROC) curves of the three divisions are plotted in Fig. 6. We also reported the equal error classification rate (EER) and the area under the ROC curve (AUC) as threshold-independent measures from the ROC curve. EER is defined as the classification rate where misclassification rates for both AD and NC subjects are equal. The ROC curves on the three divisions demonstrate that our method outperforms the Toews' method, with average AUC values higher 0.03. The curves also have similar trends under the effects of age and the severity of clinical diagnosis. Maximum classification rate is achieved for subjects with mild AD and within 60–80 years of age, and including wider range of age or severity of clinical diagnosis both result in reduced classification performance. On the other hand, the M3 procedure leads to much lower classification performance, indicating that the secondary set is necessary to improve the parameter estimation of specific morphological features. The results reveal that the effective correspondences and the morphological differences identified from the secondary feature sets are reliably related to the AD groups.

## Discussion

This paper presents and validates MEACOLP, a new morphometry method for detecting group-related structures in volumetric images. Correspondences of underlying anatomical structures are identified from distinctive local features to quantify the statistical regularity. The primary difference between MEACOLP and other morphometry techniques is that MEACOLP explicitly addresses the effectiveness as well as the accuracy of the correspondences for a specific morphometric analysis. A clinical validation has been performed on a set of 198 NC and probable AD subjects from the public OASIS dataset [24]. Experimental results demonstrate that MEACOLP improves both the effectiveness and the accuracy of the correspondences by using the proposed 2L-SIFT instead of the standard SIFT. Based on the improved correspondences, group-related anatomical structures known to be affected by AD are automatically discovered and well-characterized as specific morphological differences. In addition, MEACOLP is potentially useful for computer-aided diagnosis, and leave-one-out classification trials demonstrate that MEACOLP outperforms the recent FBM method [13]. The classification performance would be much lower if the secondary set from the 2L-SIFT is not used. These results reveal that the secondary set of the 2L-SIFT is effective for specific morphometric analysis, and the morphological differences are reliably related to the group of interest.

MEACOLP and FBM [13] are both based on local features, and are proposed for the situation where the inter-subject registration of underlying structures is ambiguous or difficult to achieve. However, most of the group-related patterns identified by MEACOLP are not the same as those patterns reported in FBM. For the most significant AD-related structures that are located in brain hemispheres, MEACOLP identifies majority of the structures (eight out of the ten) in only a single hemisphere while FBM identifies minority of them (two out of the eight). This demonstrates the difference between morphometric analysis based on the feature occurrence (FBM) and that based on the specific feature properties (MEACOLP), and the results of MEACOLP suggest that the specific feature properties statistically significant AD-related structures are primarily asymmetrical in nature. Two reasons are responsible for the difference. Firstly, the bases for morphometric analysis are different. MEACOLP uses the stable template patterns whose correspondences should be robustly identified in most subjects, and FBM uses a large number of model features whose correspondences are often fragment in a minority of training subjects. That is, most of model features would not be treated as template patterns. For instance, the temporal horns of the lateral ventricles are identified as group-related in FBM [13]; however, they are not stable enough to become a template pattern. Secondly, the morphological features for MEACOLP are scales and locations of the correspondences, while those for FBM are the feature occurrences. The group-related patterns of MEACOLP are caused by scaling or translation of the underlying structures. In contrast, the group-related patterns of FBM could be caused by various morphological distortions, so it is difficult to determine what kind of specific differences has happened. In conclusion, MEACOLP and FBM are two complementary morphometry methods, since MEACOLP could be used to quantify specific morphological differences for stable patterns, and FBM could be used to analyze local patterns which only occur in minority. FBM has identified some anatomical patterns that are present primarily in a single subject group. Such patterns could not be identified directly in MEACOLP. However, these patterns might be reflected on translation of its neighboring patterns, and thus be identified indirectly by analyzing the geometric properties of their neighboring template patterns.

A limitation of this study is that the number of template patterns is not sufficient for discovering more group-related structures, especially in regions of highly variable cortices. Some cortices are shown to be group-related in previous studies [13]; however, they are not detected in MEACOLP. Patterns fail to generate template patterns mainly due to the ambiguous situations where the same underlying cortices vary from subject to subject yet different underlying cortices look alike. Although a geometry constraint has been applied to reduce the ambiguous situations, it is not strict enough for the cortical regions. Our future work will include performing more sophisticated techniques to generate template patterns in cortical regions. In particular, we plan to compare the performances of various description methods for local features, e.g. histogram of gradients [18], in order to extract more distinctive appearance descriptors. Moreover, the topology of local features [17] and non-linear registration which can align potential correspondences with small registration error [23], [34] will be taken into consideration to enhance the geometry constraint. Finally, the influences of the preprocessing steps in OASIS dataset, i.e. averaging of multiple images and skull stripping, were not taken into account in current study. In the future, we will consider the influences, and further improve the current method for clinical analysis of neurological diseases.
